# Supplementary material for: Double-strand breaks induce inverted duplication chromosome rearrangements by a DNA polymerase δ-dependent mechanism
Source: Nat Commun. 2023 Nov 2;14:7020. doi: 10.1038/s41467-023-42640-5 (PMC10622511; doi:10.1038/s41467-023-42640-5)

Supplementary Table 1. Survival and inverted duplication frequencies

Relevant genotype	gRNA	Target IR	Median survival [a]	Relative survival [b]	Fraction inverted duplications	Inverted duplication frequency [c]	Relative inverted duplication frequency
sae2Δ	gRNA-17	Natural	0.081	1	0.91	0.074	1
mre11-H125N	gRNA-17	Natural	0.178	2.18	ND	ND	ND
mre11Δ	gRNA-17	Natural	0.093	1.14	0.87	0.081	1.08
WT	gRNA-17	Natural	0.0004	0.0048	0.09	3.54×10^{-5}	4.7×10^{-4}
sae2Δ	gRNA-17	Scrambled	0.0035	0.044	0.16	0.0006	0.008
sae2Δ rad1Δ	gRNA-17	Natural	0.087	1.07	0.86	0.075	1.01
sae2Δ pol3-01	gRNA-17	Natural	0.0068	0.084	0.65	0.004	0.06
sae2Δ pol3-01 rad1Δ	gRNA-17	Natural	0.0039	0.048	0.76	0.003	0.04
sae2Δ mus81Δ	gRNA-17	Natural	0.089	1.01	0.87	0.077	1.04
sae2Δ	gRNA-17	Perfect	0.102	1.25	0.93	0.095	1.28
sae2Δ rad1Δ	gRNA-17	Perfect	0.308	3.79	0.93	0.288	3.97
sae2Δ pol3-01	gRNA-17	Perfect	0.016	0.20	0.41	0.007	0.09
sae2Δ pol3-01 rad1Δ	gRNA-17	Perfect	0.026	0.32	0.42	0.011	0.15
sae2Δ	gRNA-48	Natural	0.011	0.13	0.59	0.006	0.087
sae2Δ rad1Δ	gRNA-48	Natural	0.0082	0.10	0.53	0.004	0.059
sae2Δ	gRNA-160	Natural	0.0017	0.021	0.48	0.0008	0.011
sae2Δ rad1Δ	gRNA-160	Natural	0.0013	0.016	0.15	0.0002	0.003
sae2Δ pol32Δ	gRNA-17	Natural	0.0039	0.048	0.13	0.0005	0.007
sae2Δ rad51Δ	gRNA-17	Natural	0.0029	0.0357	0.83	0.002	0.032
sae2Δ	HO	Natural	0.264	3.25	0.92	0.24	3.28
WT	HO	Natural	0.0033	0.012	0.025	8.26×10^{-5}	0.001

[a] Median survival value from at least 6 independent cultures
[b] Cell survival normalized to the *sae2Δ* mutant with the natural IR and gRNA-17
[c] Frequency of inverted duplications was derived from the fraction of survivors with a CNV value of >1.5.

Supplementary Table 2. Analysis of survivors using gRNA-48 and gRNA-160

**Strain**	**N**	**Inverted duplications**	**Target IR**	**Non-target IR (telomeric)**	**Non-target IR (centromeric)**
*sae2*Δ gRNA-48	60	38	27	3	8
*sae2*Δ *rad1*Δ gRNA-48	60	40	17	21	2
*sae2*Δ gRNA-160	110	52	6	12	34
*sae2*Δ *rad1*Δ gRNA-160	104	28	7	11	10

Supplementary Table 3. Yeast Strains

Strain	Relevant Genotype[1]	gRNA	IR	Nuclease
W1588-4C	*MAT***a**	n/a	Native	n/a
LSY4708	*MAT***a**	n/a	Native	P_{lexO}-Cas9-ER
LSY4713	*MAT***a**	gRNA-17	Native	P_{lexO}-Cas9-ER
LSY4714	*MATα*	gRNA-17	Native	P_{lexO}-Cas9-ER
LSY4751-11C	*MATα sae2::KanMX6*	gRNA-17	Native	P_{lexO}-Cas9-ER
LSY4752-4C	*MATα mre11::HIS3MX6*	gRNA-17	Native	P_{lexO}-Cas9-ER
LSY4763-7D	*MAT***a** *mre11-H125N*	gRNA-17	Native	P_{lexO}-Cas9-ER
LSY4903	*MAT***a**	gRNA-17	Scrambled	P_{lexO}-Cas9-ER
LSY4913-2D	*MATα sae2::KanMX6*	gRNA-17	Scrambled	P_{lexO}-Cas9-ER
LSY4616	*MAT***a** *rad1::HphMX6*	n/a	Native	n/a
LSY4762-6A	*MATα rad1::HphMX6*	gRNA-17	Native	P_{lexO}-Cas9-ER
LSY4762-45D	*MATα sae2::KanMX6 rad1::HphMX4*	gRNA-17	Native	P_{lexO}-Cas9-ER
LSY5068-8D	*MAT***a** *sae2::KanMX6 pol3-01*	gRNA-17	Native	P_{lexO}-Cas9-ER
LSY5068-14D	*MATα sae2::KanMX6 pol3-01 rad1::HphMX4*	gRNA-17	Native	P_{lexO}-Cas9-ER
LSY5052	*MAT***a**	gRNA-48	Native	P_{lexO}-Cas9-ER
LSY5054	*MATα sae2::KanMX6*	gRNA-48	Native	P_{lexO}-Cas9-ER
LSY5056	*MAT***a** *rad1::HphMX4*	gRNA-48	Native	P_{lexO}-Cas9-ER
LSY5193	*MATα sae2::KanMX6 rad1::HphMX4*	gRNA-48	Native	P_{lexO}-Cas9-ER
LSY5053	*MAT***a**	gRNA-160	Native	P_{lexO}-Cas9-ER
LSY5055	*MATα sae2::KanMX6*	gRNA-160	Native	P_{lexO}-Cas9-ER
LSY5057	*MAT***a** *rad1::HphMX4*	gRNA-160	Native	P_{lexO}-Cas9-ER
LSY5059	*MAT***a** *sae2::KanMX6 rad1::HphMX4*	gRNA-160	Native	P_{lexO}-Cas9-ER
LSY4904	*MAT***a**	gRNA-17	Perfect	P_{lexO}-Cas9-ER
LSY5171-23B	*MATα sae2::KanMX6*	gRNA-17	Perfect	P_{lexO}-Cas9-ER
LSY5171-44C	*MATα sae2::KanMX6 rad1::HphMX4*	gRNA-17	Perfect	P_{lexO}-Cas9-ER
LSY5171-46A	*MAT***a** *sae2::KanMX6 pol3-01*	gRNA-17	Perfect	P_{lexO}-Cas9-ER
LSY5235-1	*MATα sae2::KanMX6 pol3-01 rad1::HphMX4*	gRNA-17	Perfect	P_{lexO}-Cas9-ER
LSY5169	*MATα rad51::NatMX4*	gRNA-17	Native	P_{lexO}-Cas9-ER
LSY5000-6D	*MAT***a** *sae2::KanMX6 rad51::NatMX4*	gRNA-17	Native	P_{lexO}-Cas9-ER
LSY4880	*MAT***a** *pol32::NatMX4*	gRNA-17	Native	P_{lexO}-Cas9-ER
LSY4999-3A	*MAT***a** *sae2::KanMX6 pol32::NatMX4*	gRNA-17	Native	P_{lexO}-Cas9-ER

LSY5750-6C	*MAT***a** *mus81::HphMX4*	gRNA-17	Native	P_{lexO}-Cas9-ER
LSY5751	*MAT*α *sae2::KanMX6 mus81::HphMX4*	gRNA-17	Native	P_{lexO}-Cas9-ER
LSY5732-37D	*MAT***a***-inc hml*Δ *hmr*Δ *leu2::P-GAL-HO-LEU2 ChrV-32878-32897::HOcs*	HOcs	Native	P_{GAL}-HO
LSY5744-2A	*MAT***a***-inc hml*Δ *hmr*Δ *leu2::P-GAL-HO LEU2 ChrV-32878-32897::HOcs sae2::KanMX6*	HOcs	Native	P_{GAL}-HO

Supplementary Table 4. Oligonucleotides

Oligos used to make gRNA constructs

Oligo	Sequence
RF-gRNA-S	20xNGTTTTAGAGCTAGAAATAGCAAGT
RF-gRNA-AS	20xNAAAGTCCCATTCGCCACCCGAAGG
pCeASY-gRNA-S	ttt20xNgttttagag
pCeasy-gRNA-AS	CTAGctctaaaac20XNAaaa

The sequences of gRNAs used in this study

Oligo	Sequence	Coordinates[1]
gRNA-17	ACGTCCAAAATTGAATGACT	ChrV:32878-32897
gRNA-48	ATACTAATCCATGCCGCCAG	ChrV:32847-32866
gRNA-160	GTATATTAGAAACCCGATAA	ChrV:32735-32754
gRNA_mut1	TTGGACGTACAAAGTTCCAC	ChrV:32885-32866
gRNA_mut2	GCCAACTACACTAAGTTCCA	ChrV:32900-32919
pol3-01	ACCAGCACACTCGATATCAA	ChrIV:277840-277830

[1]The coordinate listed are based on the S288C reference genome

Oligos use to make the repair templates for CRISPR-Cas9 gene editing

Oligo	Sequence
IR_500_upstream_F	TGTTAGTTTAGGGTCATTGTAT
IR_500_downstream_R	GAAGCATATGTACAATGAGC
Left_IR_perfect_R	AATCACTTTTGCCCaaGAACTTAGTGTAGT
Right_IR_perfect_F	ACTACACTAAGTTCttGGGCAAAAGTGATT
Left_scrambledIR_R	ttTGCAttCGatCTCcAtATTcAGCGgGCTTAGTGTAGTTGGCCAAGTC
Right_scrambledIR_F	CcCGCTgAATaTgGAGatCGaaTGCAaaAACCAATACATGTAACCATTG
pol3-01_middle	ATCCTACCAGCACACGCGATAGCAAAGGACATGATACGCAATGGAGCTGT
pol3-01_left	CCTAATAGCACATCCTGCTGAGGGTGATTGGTCTCATACAGCTCCATTGC
pol3-01_right	GTGTGCTGGTAGGATTGGCGTCTTTCCGGAACCTGAATACGATCCCGTCA
pol3-01_mut_F	GTTTCAATTAATTATCGTAACCTAATAGCACATCCTGCTG
pol3-01_mut_R	CACAACGTTGGCAATTTGGATGACGGGATCGTATTCAGG
HOcs_gRNA2_mut_F3	AGTTTCAGCTTTCCGCAACAGTATAATTTTATAAACAAGTTCCAGGGCAAaagtgat
HOcs_gRNA2_mut_R3	GTTGCGGAAAGCTGAAACTAAAGTTCCACTGGCGGCA

Oligos used for screening survivors

Oligo	Sequence	Coordinates[1]
P1	CATCACCCTGTGCGTTTTACAAG	ChrV:8772-8794
P2	AATGAGTCAGCTGGATCTATTGCT	ChrV:9199-9176
P3	AGGAAACCCAACCTAAGAAC	ChrV:32602-32621
P4	GTGGGCGCTCTTATATCAT	ChrV:33109-33091
P5	CGGGTGTTATGCCAACGTTG	ChrV:60074-60093
P6	GGCAACTGTCTACCTATTTCCAT	ChrV:60140-60118
P7	TGATGGCTCTTGGAACGGAT	ChrV:32503-32522
P8	CGCTCTTTCCCGACGAGA	ChrV:33310-33293

Other oligos

Oligo	Sequence	Coordinates
P9 (ADH1 F)	TAAGGGCTGGAAGATCGGTGAC	ChrXV:160355-160334
P10 (ADH1 R)	CGTCGTGGGTGTAACCAGACA	ChrXV:160225-160245
P11 (17bp upstream F)	AATGACTTGGCCAACTACAC	ChrV:32891-32910
P12 (17bp downstream R)	TTTATCCACACCTCTGACCAACG	ChrV:33140-33118
P13 (CAN1 control F)	GACCTGTACCAATAGTACCACC	ChrV:33151-33172
P14 (CAN1 control R)	ACAGAGTAAACCGAATCAGGG	ChrV:33677-33657
P15 (MEC1 control F)	GAGTACAGGCATGTGATGT	ChrII:504971
P16 (MEC1 control R)	CCGAGTTCAGGTCTTTTATTGC	ChrII:505728

Supplementary Table 5. Plasmids used in this study

Plasmid	Description	Marker	Source
pML107	P_{TDH3}-*CAS9-NLS* with gRNA expression cassette	*LEU2*	(Laughery, Hunter et al. 2015)
pAA1	P_{GAL1}-*CAS9-NLS* with gRNA expression cassette with BaeI site for gRNA cloning	*LEU2*	This study
pAA3	P_{GAL1}-*CAS9-NLS-FLAG* in pRG203MX backbone	*HIS3*	This study
pCAS	P_{GAL1}-*CAS9-NLS* with $P_{tRNA-LYS}$-*HDV_ribozyme-sgRNA*-T_{sNR52}	*Kan*	(Ryan, Poddar et al. 2016)
pCeASY	P_{GAL1}-*CAS9-NLS* with $P_{tRNA-LYS}$-*HDV_ribozyme-sgRNA*-T_{sNR52}, with ZraI-XbaI gRNA cloning sites	*Kan*	R. Gnügge
pAA9	$P_{tRNA-LYS}$-*HDV_ribozyme-sgRNA*-T_{sNR52}, with ZraI-XbaI gRNA cloning sites in pRG205MX backbone	*LEU2*	This study
pRG634	*lexO-HO* in pRG205MX background	*LEU2*	R. Gnügge
pRG635	P_{ACT1}-*LEXA-ER-B112*-T_{CYC1} in pRG203MX background	*HIS3*	R. Gnügge
pAA12	*lexO-linker*-T_{ADH1} P_{ACT1}-*LEXA-ER-B112*-T_{CYC1}	*HIS3*	This study
pAA13	$P_{tRNA-LYS}$-*HDV_ribozyme-sgRNA17*-T_{sNR52} in pRG205MX	*LEU2*	This study
pAA16	*lexO-CAS9-NLS-FLAG*-T_{ADH1} P_{ACT1}-*LEXA-ER-B112*-T_{CYC1} in pRG203MX	*HIS3*	This study
pAA18	*lexO-CAS9-ER-NLS-FLAG*-T_{ADH1} P_{ACT1}-*LEXA-ER-B112*-T_{CYC1} in pRG203MX	*HIS3*	This study
pAA19	*lexO-CAS9-NLS-FLAG*-T_{ADH1} P_{ACT1}-*LEXA-ER-B112*-T_{CYC1} in pRG203MX (CAS9 and LEXA-ER-B112 tail to tail)	*HIS3*	This study
pAA20	*lexO-CAS9-ER-NLS-FLAG*-T_{ADH1} P_{ACT1}-*LEXA-ER-B112*-T_{CYC1} in pRG203MX (CAS9 and LEXA-ER-B112 tail to tail)	*HIS3*	This study
pAA21	$P_{tRNA-LYS}$-*HDV_ribozyme-sgRNA48*-T_{sNR52} in pRG205MX	*LEU2*	This study
pAA22	$P_{tRNA-LYS}$-*HDV_ribozyme-sgRNA160*-T_{sNR52} in pRG205MX	*LEU2*	This study
pAA23	P_{GAL1}-*CAS9-NLS* with $P_{tRNA-LYS}$-*HDV_ribozyme-sgIR-gRNA_mut2*-T_{sNR52}	*Kan*	This study
pAA24	P_{GAL1}-*CAS9-NLS* with $P_{tRNA-LYS}$-*HDV_ribozyme-sgRNApol3-01*-T_{sNR52}	*Kan*	This study

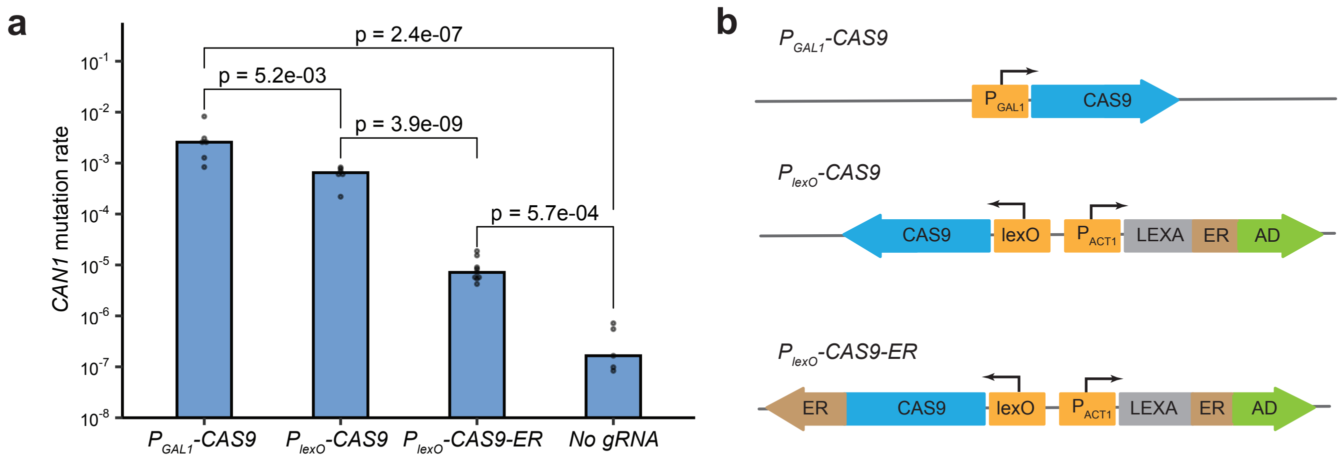

Supplementary Figure 1. Leaky expression of Cas9. a, Spontaneous mutation rate of *CAN1* in WT cells carrying the indicated the Cas9 constructs with a gRNA targeting the *CAN1* locus or without gRNA. Cas9 expression was not induced during the experiment. P values were determined using a two-tailed t-test. *PGAL1-CAS9*, *n*=6; *Plex0-CAS9*, *n*=6; *PlexO-CAS9-ER*, *n*=9; no gRNA, *n*=5; 2-4 biological replicas. **b**, Constructs used to express Cas9. Source data are provided as Source Data Supplementary Figure 1.

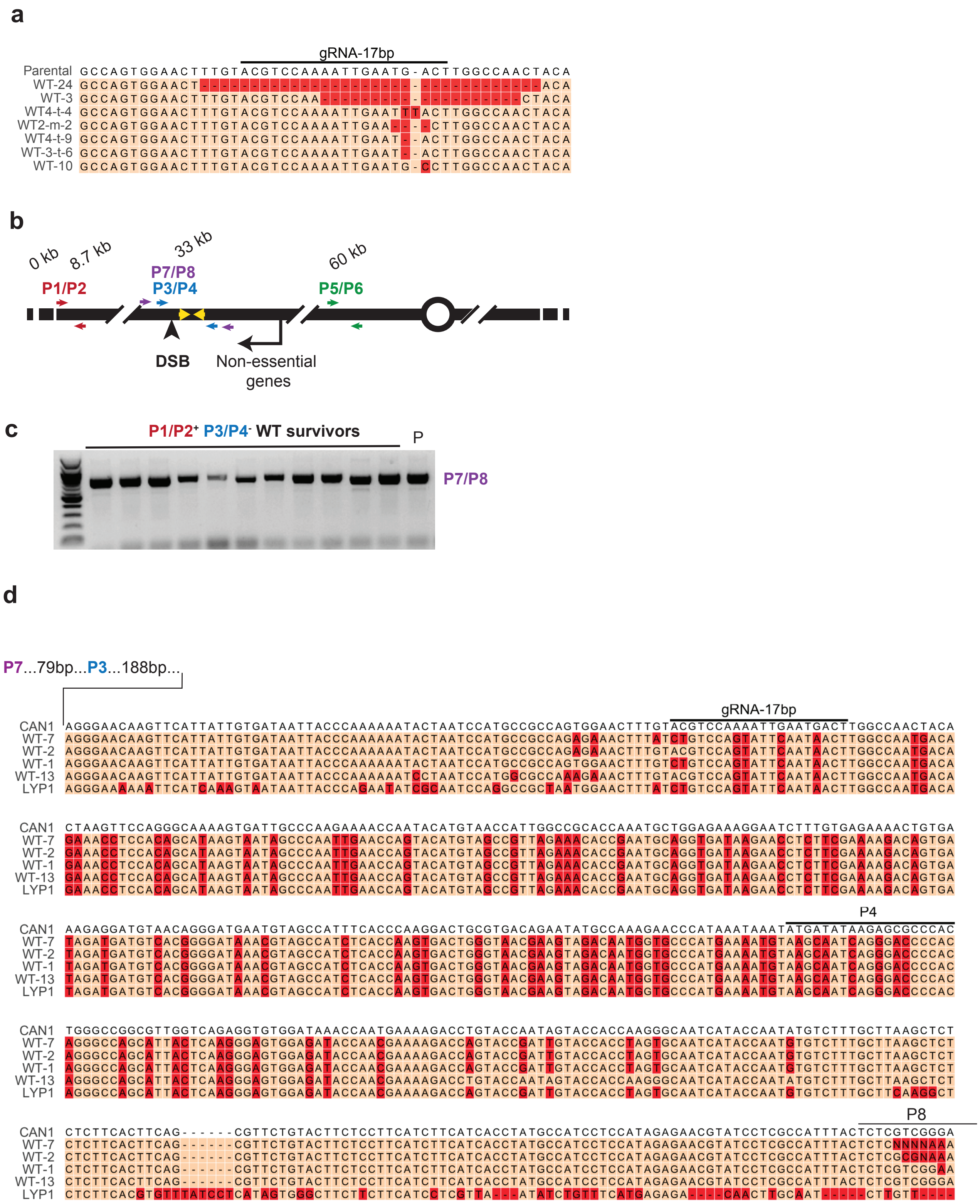

Supplementary Figure 2. Molecular analysis of survivor types from WT cells. a, The P3/P4 primer PCR products from select WT clones were sequenced and show evidence of indels. **b**, Schematic of the left arm of Chr V showing the location of the DSB and inverted repeats (yellow arrows). Primer pairs to detect retention of terminal sequence, NHEJ events and *LYP1* conversions are shown in red, blue and purple, respectively. **c**, Clones that had P1/2 but not P3/4 PCR bands were amplified using the P7/P8 primer pair to detect the presence of sequences further away from P3/P4. **d**, Sanger sequence of P7/P8 primer PCR products shows evidence of *LYP1* conversions.

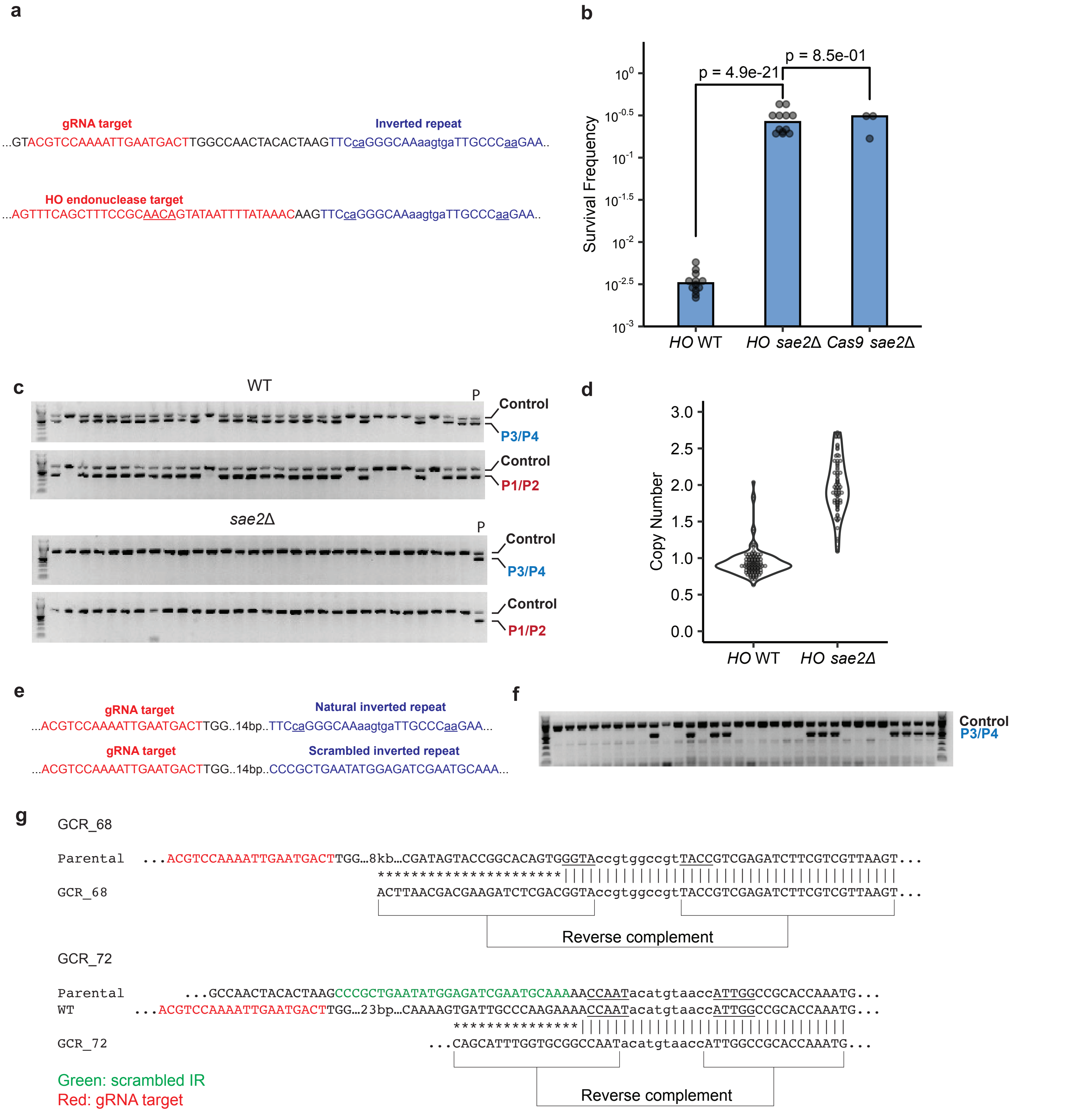

Supplementary Figure 3. HO-induced inverted duplications and analysis of survivors from the scrambled IR strain. **a**, Sequence of the original gRNA target (top) and the HO cut site that replaces it (bottom). In both, red denotes the nuclease recognition sequence. Underlined in red: the HO cut site. In purple, the inverted repeat sequence. **b**, Survival frequencies of WT and *sae2Δ* either expressing HO or Cas9. P values were determined using a two-tailed t-test. WT HO, *n*=12; *sae2Δ* HO, *n*=12; *sae2Δ Cas9, n*=3; 3 biological replicas except *sae2Δ* Cas9. **c**, Colony PCR using the indicated primers of cells surviving a DSB by HO adjacent to the IR. **d**, Copy number analysis by qPCR (primers P5/P6) of independent clones with the indicated genotypes surviving the HO-induced DSB. **e**, Sequence of the original IR and scrambled IR located 17 bp from the gRNA target sequence. **f**, The sequence at the center of the inverted duplications of two *sae2Δ* clones with the scrambled IR. Mismatches in the inverted repeat are shown in lower case. In red is the gRNA target sequence. For GCR_72, the position of the scrambled IR within the unrearranged sequence is shown for reference. **g**, 32 independent clones from *sae2Δ* cells with scrambled inverted repeat were analyzed using P3/4 primers to detect NHEJ events. Source data are provided as Source data Supplementary Figure 3.

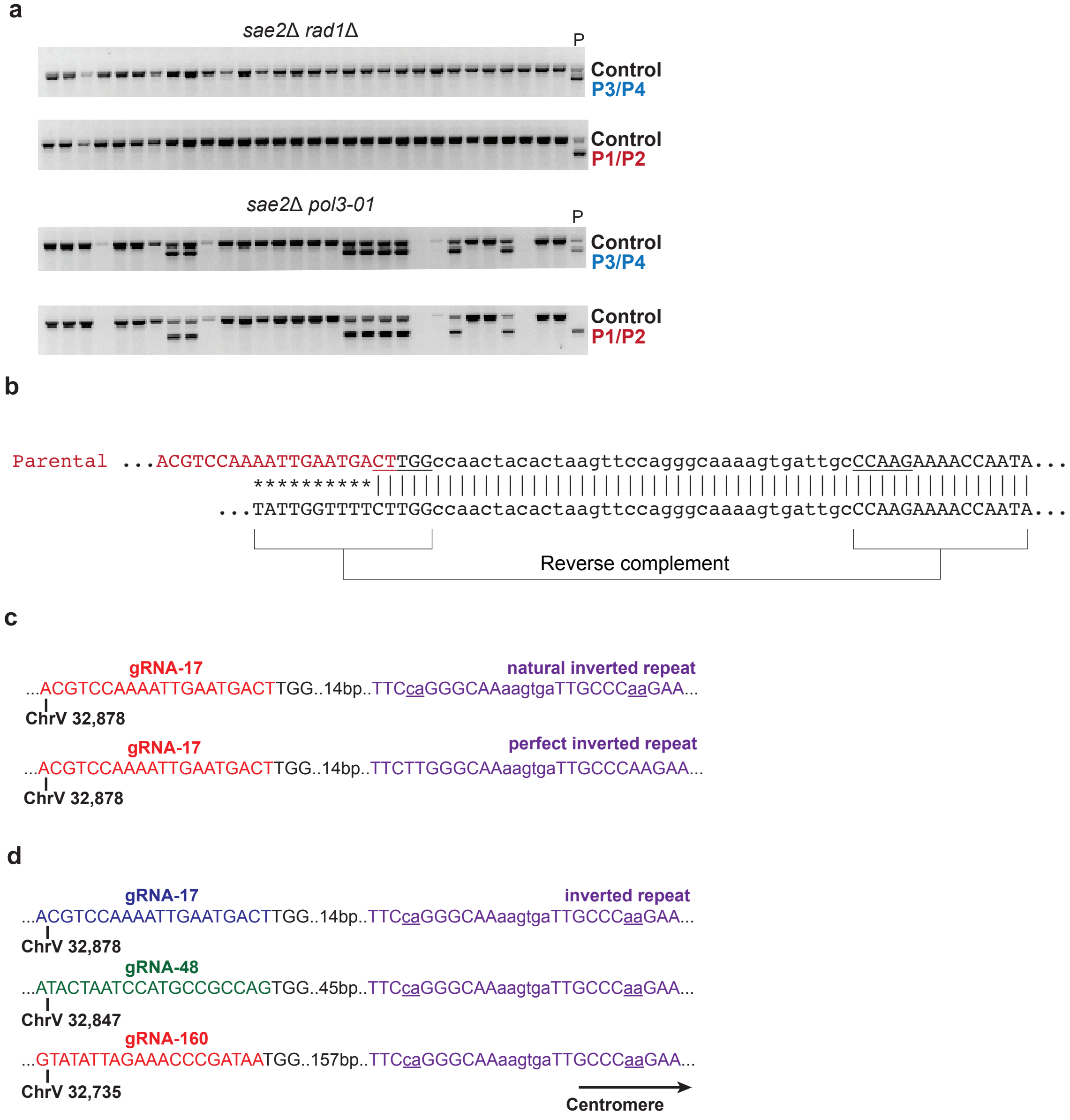

Supplementary Figure 4. Analysis of *sae2Δ rad1Δ* and *sae2Δ pol3-01* clones. **a**, 30 independent clones from *sae2Δ rad1Δ* and *sae2Δ pol3-01* cells were analyzed using P3/4 and P1/2 primers to detect NHEJ events or loss of Chr V terminal sequence. "P" denotes the parental strain. **b**, Nine inverted duplication clones from the *sae2Δ pol3-01* mutant and 11 clones from the *sae2Δ pol3-01 rad1Δ* mutant used the PAM IR directly adjacent the cut site. Note: the latter IR is the same used in the inverted duplications in WT clones (Figure 2F). **c**, The sequence of the inverted repeat (purple font) mutated to correct the mismatches (underlined, lower case). **d**, The position of the gRNA-17, gRNA-48 and gRNA-160 relative to the inverted repeat.

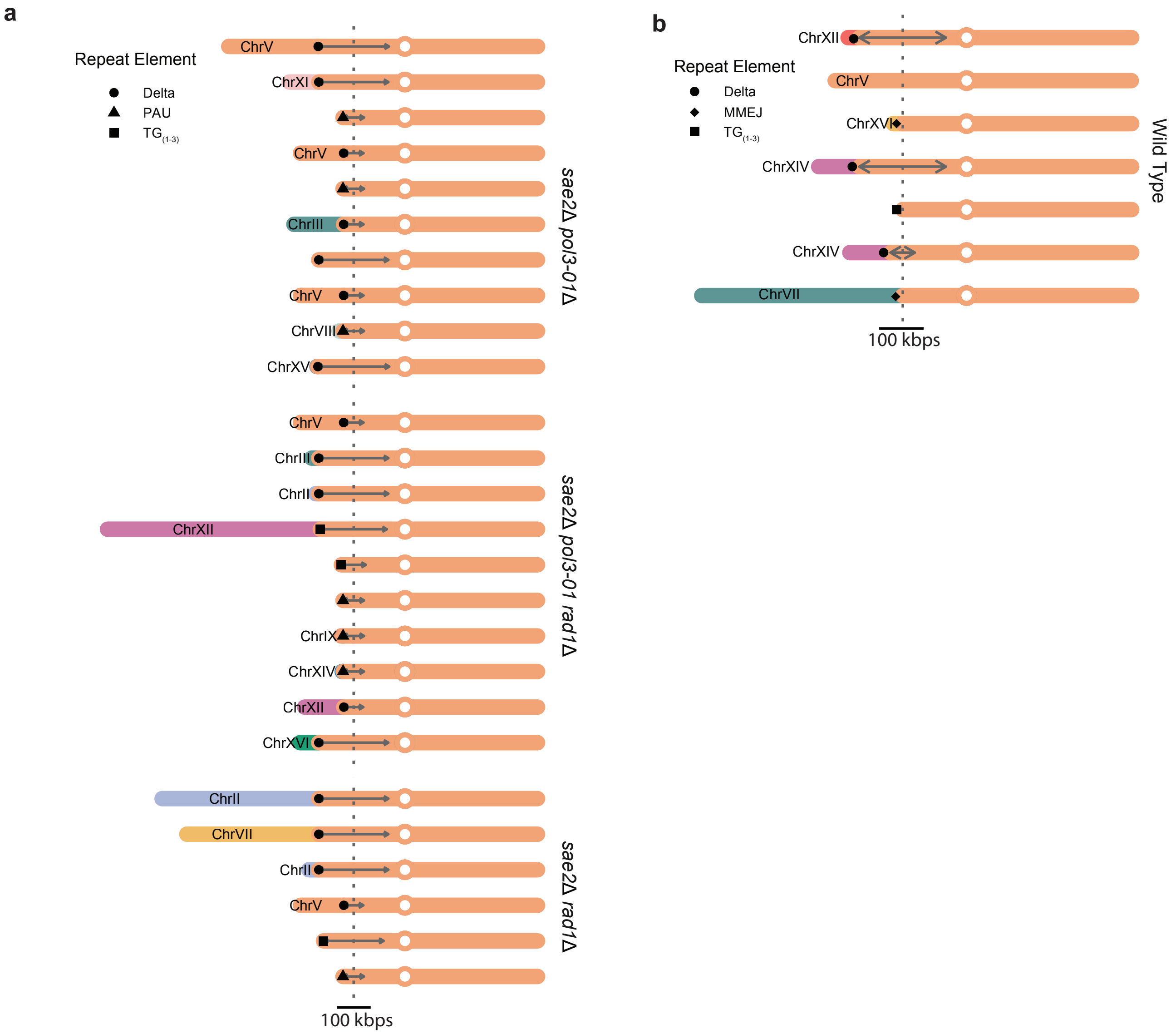

Supplementary Figure 5. NGS analysis of inverted duplication clones. a, Derivative Chr V in *sae2Δ rad1Δ, sae2Δ pol3-01* and 10 *sae2Δ rad1Δ pol3-01* clones . **b**, Derivative Chr V in WT clones, including those without inverted duplications.

a

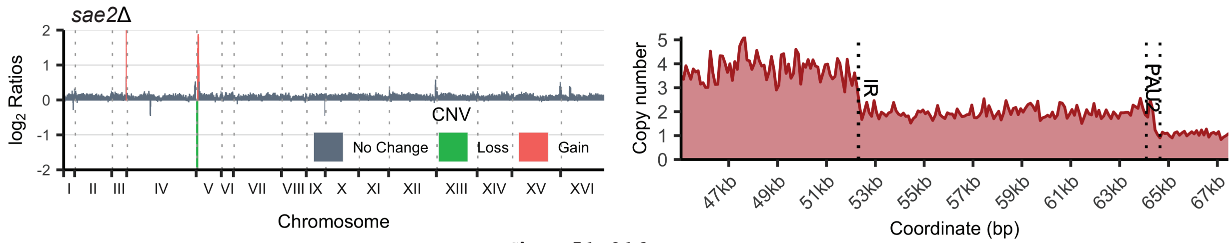

ChrV:51,916

CATAACTTGAAAAGCTGATGGGtGTcaagcACgCCCATCAGCGCATCTGAAAGTA
CATAACTTGAAAAGCTGATGGGTGTcaagcACGCCCATCAGCTTTTCAAGTTATG

b

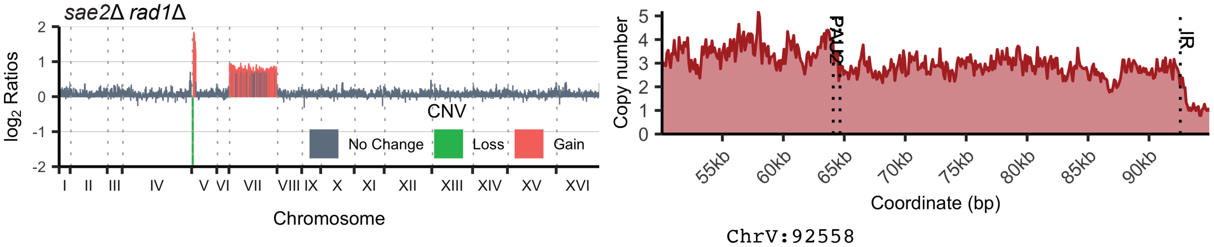

ChrV:92558

TATGCTTTGAATGCCtTggaAgGGCATTCTCAATTAA
TATGCTTTGAATGCCtTggaAgGGCATTCAAAGCATA

c

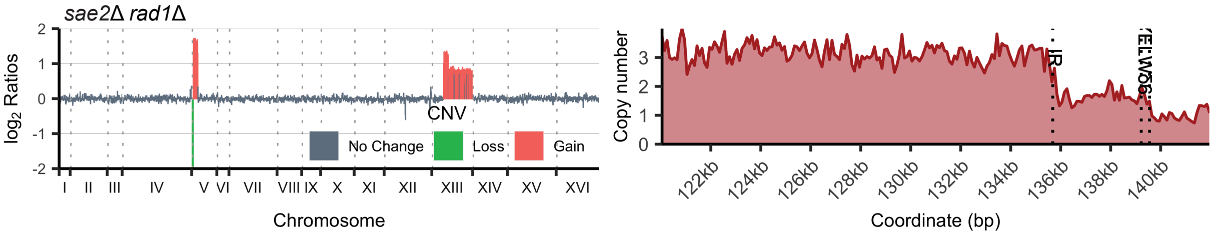

ChrV:134,684

TTGCTCTACACAAGATGATTAGGCTGATCACTCACTCGCtgggagGCGAGGCTTATTTGAACTTCGAAGGTAATGAGTTCGGTCATC
TTGCTCTACACAAGATGATTAGGCTGATCACTCACTCGCtgggagGCGAGTGAGTGATCAGCCTAATCATCTTGTGTAGAGCAA

Supplementary Figure 6. Complex rearrangements. Clones that exhibit quadruplications centromeric to the target IR in a *sae2Δ* (**a**) and in two *sae2Δ rad1Δ* clones (**b** and **c**). Left: log2 ratios of genome-wide copy number relative to parental reads. Right: relative copy numberof junctions between the higher order duplication order copy number and duplication sequence, and between the latter and non-duplication sequence. In each case, a naturally occurring IR occurs at the junction of the drop in copy number. Below each panel is the IR sequence highlighted in the right panels and occur at the center of an inverted duplication.

sae2Δ pol3-01 rad1Δ # 23

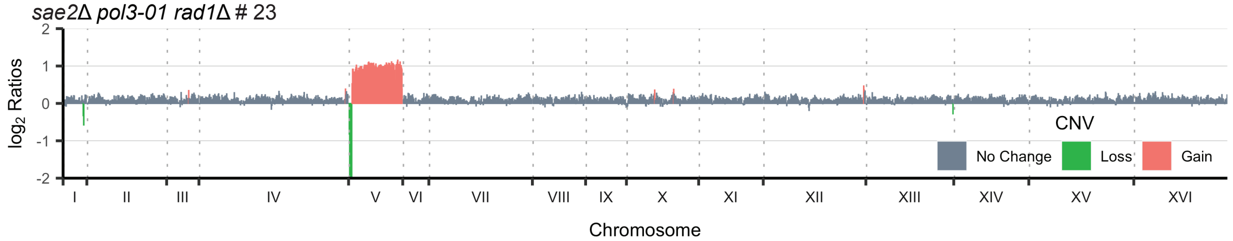


```
ChrV:151419  ...TGAATTAGGGAACGGAAGGAAAAAATCAGCAAACAGT...1.2kbp...ATGGTAGATGACTCTATAATCGTG...
                |||||||||||||||||||||||||||||||                        ||||||||||||||||||
                TGAATTAGGGAACGGAAGGAAAAAATCAGCA------------------------GATGACTCTATAATCGTG
```

sae2Δ pol3-01 rad1Δ # 25

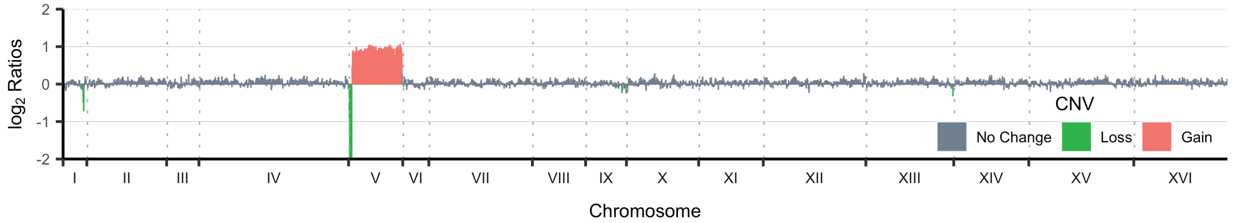


```
ChrV:151527  ...CACCGTACTTCTTTTCAATGCGTAAACAACTAGAGTTG...0.9kbps...TAGCTATTCAATGTCTGTATTCATC...
                ||||||||||||||||||||||||||||||||                         |||||||||||||||||||
                CACCGTACTTCTTTTCAATGCGTAAACAACTA-------------------------TTCAATGTCTGTATTCATC
```

sae2Δ pol3-01 rad1Δ # 27

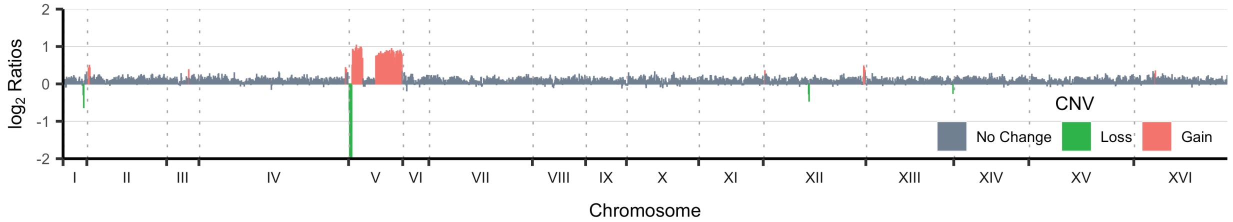


```
ChrV:147796  ...TTCCATAACATTACTTGTAAGAAAATTTATAAG...134kbp...GAAATTAAGGCCAAAGCATTGGATC...
                |||||||||||||||||||||||||||                        |||||||||||||||||||
                TTCCATAACATTACTTGTAAGAAAATT------------------------AAGGCCAAAGCATTGGATC
```

sae2Δ pol3-01 rad1Δ # 28

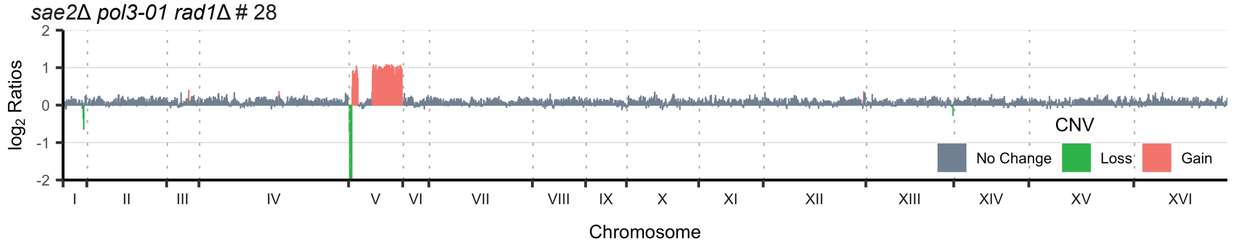


```
ChrV:96409   ...TTTCATCCTTAAGGGAATTGGTTTCTCGGAGTCAAG...150kbp...CTCTTCTCGGACCAATTTTTTGACTCTTTTCAAA...
                ||||||||||||||||||||||||||||||                            |||||||||||||||||||||||
                TTTCATCCTTAAGGGAATTGGTTTCTCGGA----------------------------CCAATTTTTTGACTCTTTTCAAA
```

sae2Δ pol3-01 rad1Δ # 29

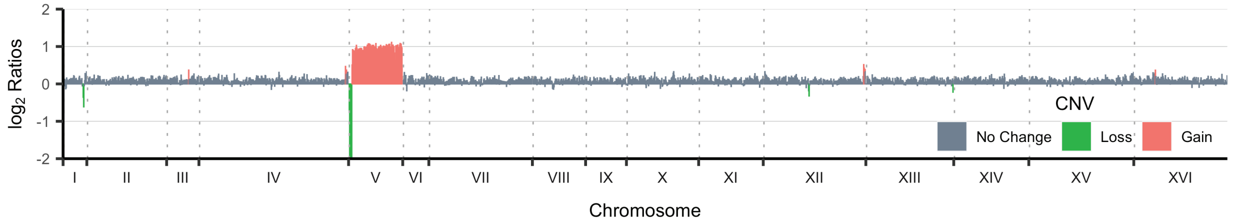


```
ChrV:151613  ...GCACTTCGTAATGTTTCTGTCATTGAATGGCG...0.8kbp...TAACTACTTTGGCTCAAAGTCAAACAAAACT...
                ||||||||||||||||||||||||||                        |||||||||||||||||||||||||
                GCACTTCGTAATGTTTCTGTCATTGA------------------------CTTTGGCTCAAAGTCAAACAAAACT
```

sae2Δ pol3-01 rad1Δ # 31

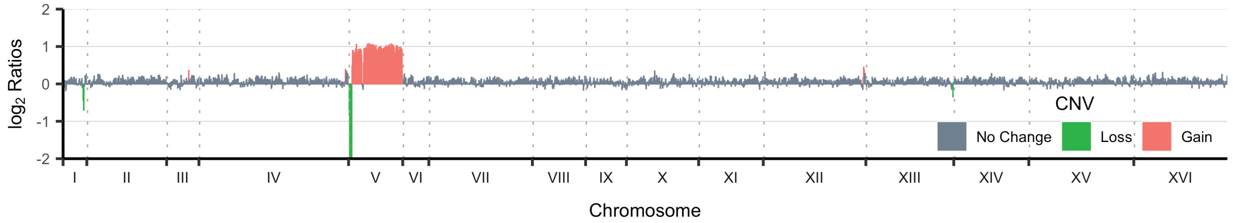


```
ChrV:135524  ...CCACTAATTTAGATGACTATTTCTCATCATTTGCG...17.5kb...GGAAGACTTAATCATTGCGAGGTCTCAATTGG...
                |||||||||||||||||||||||||||||                        |||||||||||||||||||||||||||
                CCACTAATTTAGATGACTATTTCTCATCA------------------------CTTAATCATTGCGAGGTCTCAATTGG
```

Supplementary Figure 7. Inverted duplications spanning most of the sequences centromeric to the DSB with a centromere loss. Genome-wide copy number analysis of 6 independent *sae2Δ rad1Δ pol3-01* clones that survived a DSB targeted by gRNA17. Below each copy number plot is the sequence at the junction of the rearrangement that spans a deleted *CEN5* and aligned to the parental sequence (coordinates are from the S288C genome published on SGD).

Supplementary Figure 2c

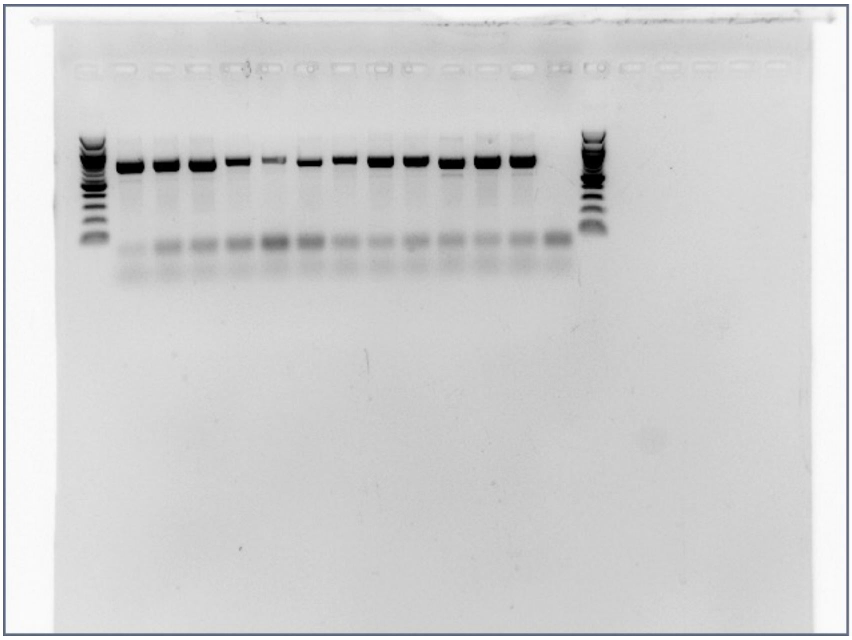

Supplementary Figure 3c

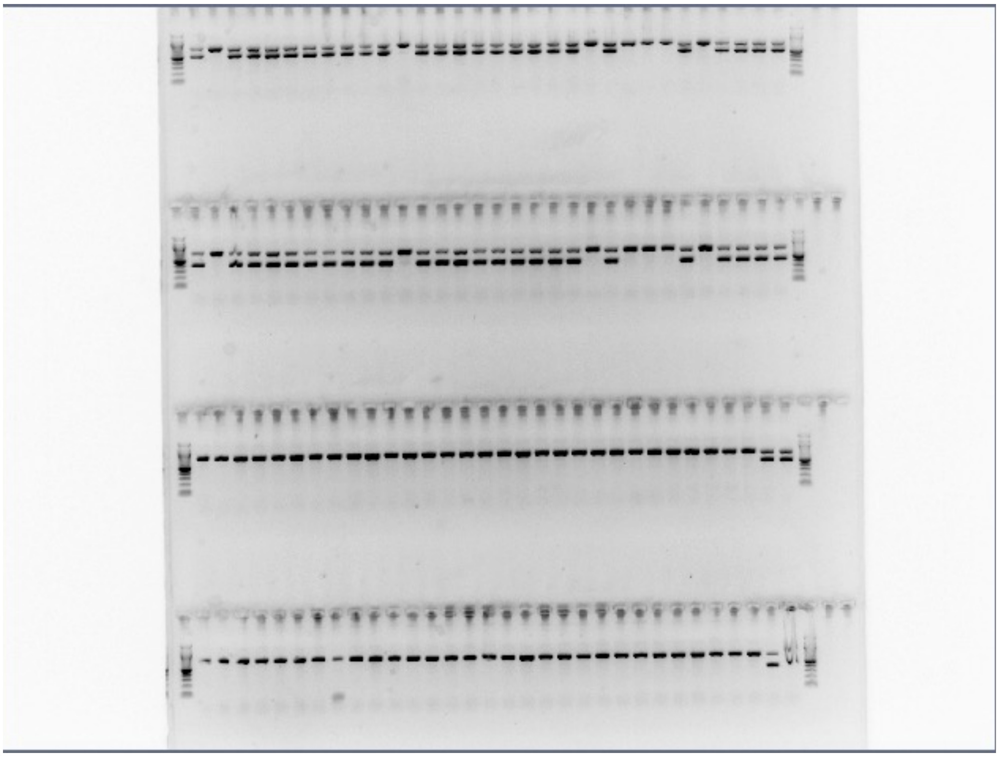

Supplementary Figure 3f

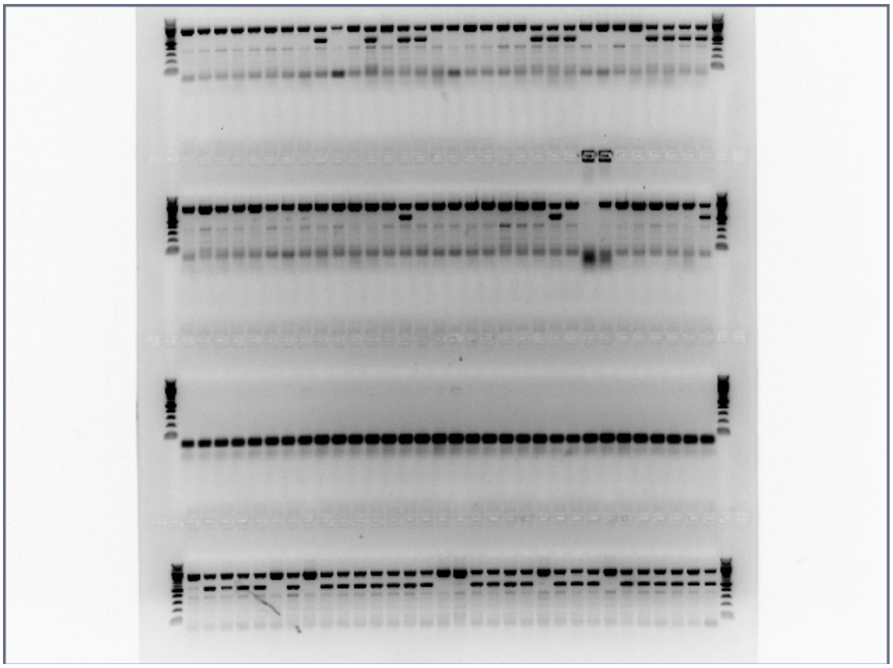

Supplementary Figure 4a

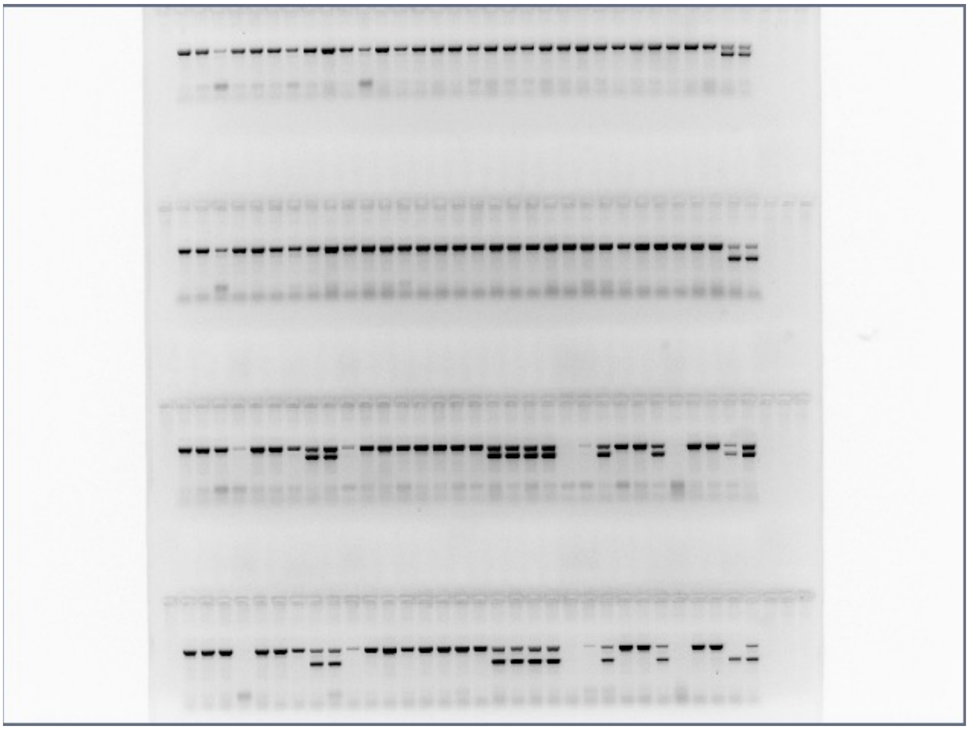

Supplement: Supplementary file 1 — Suppplementary Information [file 41467_2023_42640_MOESM1_ESM.pdf]
